# Can allelopathy of *Phragmites australis* extracts aggravate the effects of salt stress on the seed germination of *Suaeda salsa*?

**DOI:** 10.3389/fpls.2022.990541

**Published:** 2022-09-16

**Authors:** Jingwen Gao, Bo Guan, Minjia Ge, Franziska Eller, Junbao Yu, Xuehong Wang, Jincheng Zuo

**Affiliations:** ^1^Collage of Life Sciences, Ludong University, Yantai, China; ^2^The Institute for Advanced Study of Coastal Ecology, Ludong University, Yantai, China; ^3^School of Municipal and Environmental Engineering, Jilin Jianzhu University, Changchun, China; ^4^Department of Biology, Aarhus University, Ole Worms Allé 1, Aarhus, Denmark

**Keywords:** *Phragmites australis*, *Suaeda salsa*, allelopathy, seed germination, radicle length, biomass

## Abstract

*Phragmites australis* is highly adaptable with high competitive ability and is widely distributed in the coastal wetland of the Yellow River Delta. However, allelopathic effects of *P. australis* on the growth of neighboring plants, such as *Suaeda salsa*, are poorly understood. In this study, germination responses of *S. salsa* seeds collected from two different habitats (intertidal zone and inland brackish wetland) to the extracts from different part of *P. australis* were compared. Potential allelopathic effects on germination percentage, germination rate, radicle length, and seedling biomass were analyzed. The germination of *S. salsa* was effectively inhibited by *P. australis* extract. Extract organ, extract concentration, and salt concentration showed different effects, the inhibitory rates were highest with belowground extract of *P. australis* between the four different parts. Germination percentage and germination rate were significantly decreased by the interactive effect of salt stress and extract concentration in *S. salsa* from a brackish wetland but not in *S. salsa* from the intertidal zone. The impact of different extracts of *P. australis* on radicle length and seedling biomass of *S. salsa* showed significant but inconsistent variation. The response index results showed that the higher concentration of extract solution (50 g·L^−1^) of *P. australis* had stronger inhibitory effect on the seed germination and seedling growth of *S. salsa* while the belowground extract had the strongest negative effect. Our results indicated that allelopathy is an important ecological adaptation mechanism for *P. australis* to maintain a high interspecific competitive advantage in the species’ natural habitat.

## Introduction

In natural ecosystems, competition exists among plant populations, which is an important factor in shaping ecological structure at different scales (Fowler, 1986, 2013). Competition has been defined in terms of species-level traits and competitive outcomes as the ability of individuals to encroach, grab resources, or otherwise inhibit the fitness of their neighboring species (Aschehoug et al., 2016). A well-known example of resource competition is the competition for light by rapid length growth to inhibit the growth and survival of neighboring species (Craine and Dybzinski, 2013). Sometimes, competition for resources is considered to be an important driver of plant community diversity and dynamics (Viola et al., 2010). Another factor that affects the ecological patterns of plant communities is allelopathy, the chemically mediated interfering of interplant competition (Raynaud and Leadley, 2004). During allelopathy, plants can release chemicals into the environment and inhibit germination and growth of neighboring species by altering their metabolism, or by affecting their soil community symbionts (Fernandez et al., 2016). An example of a typical allelochemical is Sorgoleone, which is released through root secretions of Sorghum, disrupting several targets in the photosynthetic electron transport chain of neighboring plants (Dayan et al., 2009).

Allelochemicals are released through four pathways: leaching by rain, decomposition of plant residues, exudation from roots, and volatilization (Kulshreshtha and Gopal, 1983; Agami and Waisel, 1985; Elakovich and Wooten, 1989; Gross, 2003). Until now, experiments with plant extracts (i.e., leachates and exudates) have been regarded as an effective method to assess allelopathic effects, although the synthesis of allelochemicals in plants and their concentrations fluctuate throughout the year (Helmig et al., 2013; Santonja et al., 2018). The research on plant allelopathy mostly focuses on alien invasive species, which can affect seed germination, seedling growth, flowering and fruiting of mature bodies, and ultimately causing a decline in species diversity and eventually forming a single dominant species community (Corbin and D'Antonio, 2004). Allelochemicals can be useful as low-cost natural repressors of undesirable vegetation. For example, *Spartina alterniflora*, which is highly invasive in China, has been tested as biological resource against harmful algal blooms. The species’ extracts decreased chlorophyll a and weakened photosynthesis of the microalga *Microcystis aeruginosa* at high aqueous extract concentrations, although the allelochemical effect was beneficial for the algae at low extract concentrations (Yuan et al., 2019).

Certain highly competitive native species are able to inhibit the invasion process of alien species through allelopathy, especially if they have not co-evolved and therefore exhibit lower resistance (Callaway and Aschehoug, 2000). Thus, the native species *Sambucus ebulus* was shown to use allelopathy as a biotic containment mechanism against the naturalization of the *Fallopia x bohemica* invasive species (Christina et al., 2015). Conversely, the North American invader *Centaurea diffusa* repressed native grass species to a stronger degree than closely related grass species from communities to which *Centaurea* was native (Callaway and Aschehoug, 2000). *Phragmites australis* is a common rhizomatous graminaceous plant in wetlands. It is widely distributed in coastal marshes and inter-river depressions with shallow groundwater depth (Hellings and Gallagher, 1992; Wang et al., 2017). It has a wide ecological niche and often forms monodominance in its habitat (Zhang et al., 2003). It is also one of the most widespread wetland plants in temperate regions of the world. In the Yellow River Delta (YRD), *P. australis* is a dominant wetland species distributed from intertidal zone to inland brackish wetland. It is widely used for wetland restoration and plays an important role in the maintenance of wetland ecosystem functions of the YRD. *Phragmites australis* is an efficient competitor due to its strong root system, sexual and asexual reproduction, salt tolerance, and a wide range of edaphic niches (Guan et al., 2017, 2021). However, whether allelopathy of *P. australis*, as native species, plays an important role in the process of competition with neighboring plants is still unknown.

*Suaeda salsa* (L.) is a pioneer species in coastal wetlands of Northern China, covering most forelands in the Yellow River Delta as a part of the natural succession in coastal wetland ecosystems (Guo et al., 2020). Over the past decades, the coastal wetlands of the YRD were suffering serious ecological degradation and soil salinization due to freshwater restriction, neglect of marine areas, and climate change (Zhao et al., 2010; Yu et al., 2014). The area of *S. salsa* is also decreasing with each passing year. *Phragmites australis* is always co-occurring with *S. salsa* in the coastal wetland of the Yellow River Delta, but little is known about a potential competitive or even allelopathic effect of *P. australis* on *S. salsa*.

In this study, germination experiments were conducted to investigate the response of seed germination and early seedling growth of *S. salsa* to the extracts of different organs of *P. australis* within different salinity gradients. The purpose of the study was to solve the following questions: (1) Do the extracts of different organs of *P. australis* have negative effects on seed germination of *S. salsa*? (2) Can salt stress aggravate the potential allelopathic effect of *P. australis* on *S. salsa*? The results can provide insight into the competition mechanism of the two dominant plant communities and give valuable implications for the restoration of degraded wetlands in the Yellow River Delta.

## Materials and methods

### Study site description

The Yellow River Delta (118°33′–119°20′ E, 37°35′–38°12′ N) is located in the northern part of Shandong Province and the south coast of the Bohai Sea, a warm temperate monsoon climate zone with an average annual temperature of 12.1°C, average annual rainfall of 551.6 mm and average annual evaporation of 1,962 mm (Song et al., 2016). The dominant soil types are classified as Calcaric Fluvisols, Gleyic Solonchaks, and Salic Fluvisols (FAO), which developed on loess material carried by water from the Loess Plateau. *Phragmites australis* and *S. salsa* are dominant species in the YRD (Guan et al., 2013).

### Sample collection and treatment

#### Sample collection

**Figure 1:**
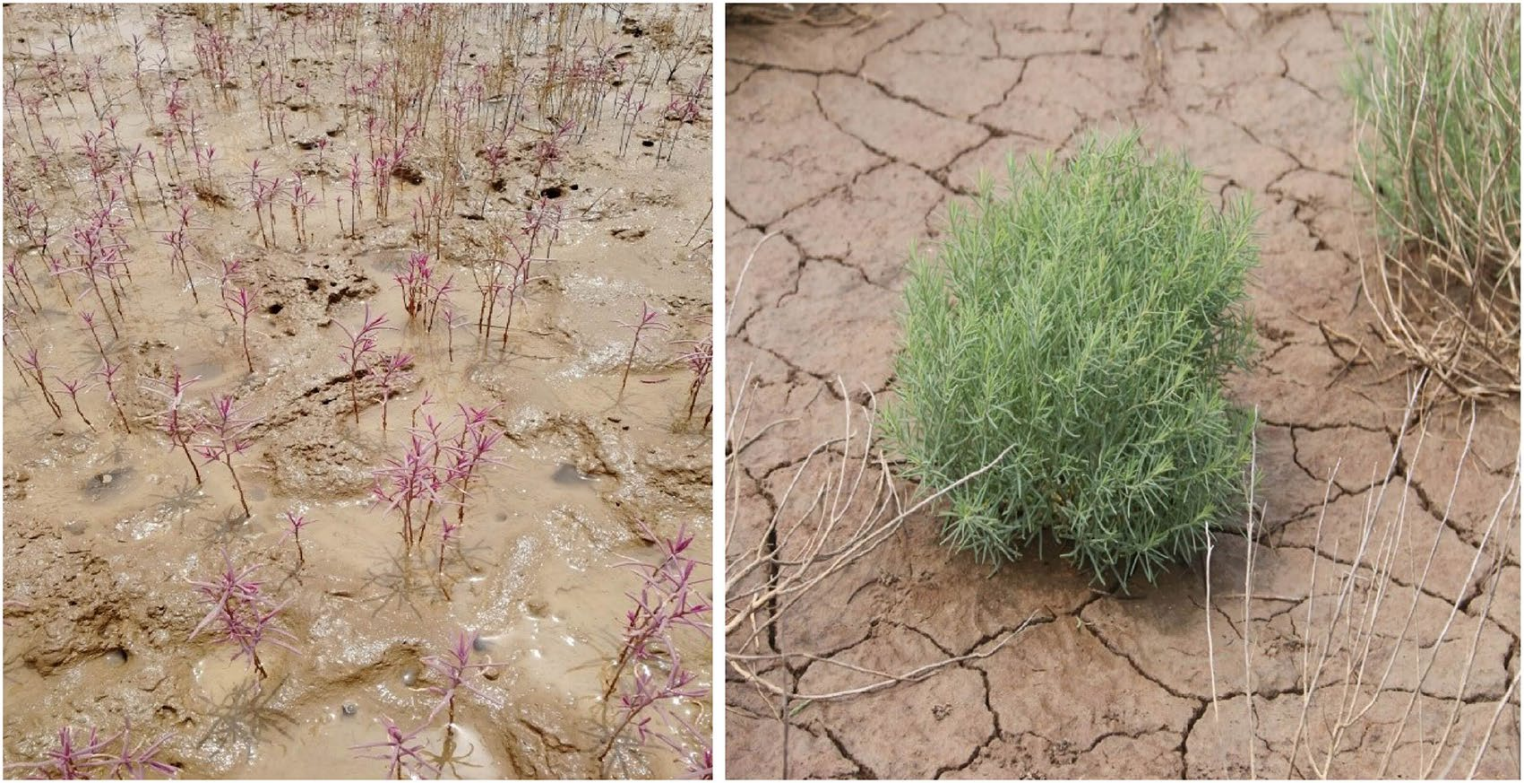
Comparison of plant morphology of *Suaeda salsa* in two habitats, left-Intertidal zone, and right-Brackish wetland.

**Figure 2:**
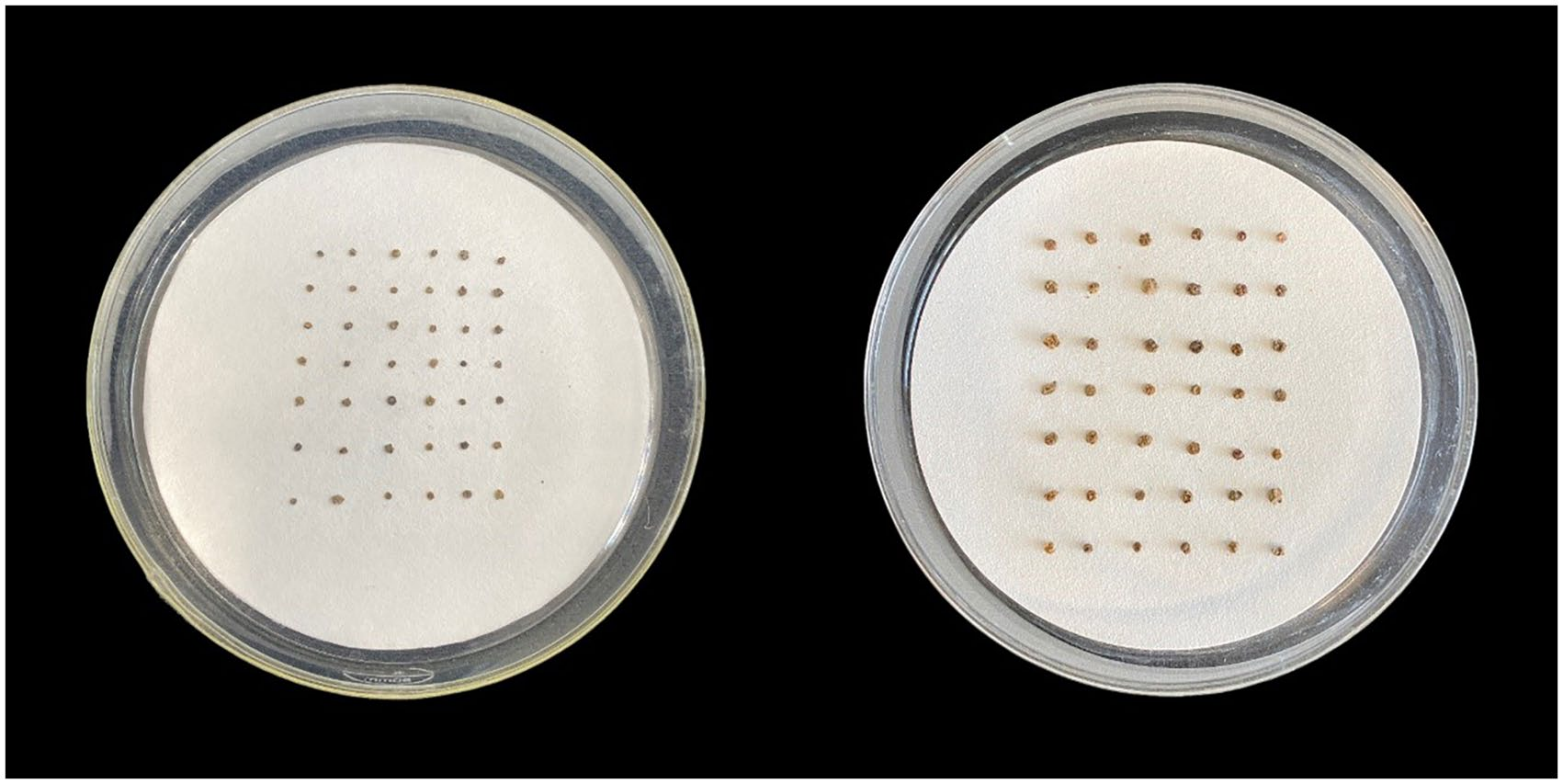
Comparison of seed size of *Suaeda salsa* in two habitats, left-Brackish wetland, right-Intertidal zone.

The seeds of two ecotypes of *S. salsa* were collected from, respectively, the intertidal zone and an inland brackish wetland of the YRD in December 2020 (Figure 1). The seed morphology and 1,000 seed weight of the two habitats were significantly different. The seeds from the intertidal zone were dark brownish-red in color and bigger, with a 1,000-seed weight of 1,453 ± 43.1 mg, while the seeds of the brackish wetland were black-brown and smaller, with a 1,000-seed weight of 376 ± 35.5 mg (Figure 2). Seeds with mature and uniform were randomly selected in this study (both commonly occurring seed-types of each *S. salsa* individual were included), and collected seeds were stored at 4°C until the experiment began.

The *P. australis* samples were collected from the same brackish wetland, which was co-inhabited by *S. salsa* in the YRD. Four parts of the *P. australis* community were collected, which were belowground biomass, aboveground biomass, litter (collected from the ground), and rhizosphere soil. The samples were washed and air dried until being used for extracts.

#### Aqueous extracts of *Phragmites australis*

The samples were crushed in a grinder and sieved through a 2 mm mesh sieve. The supernatant of the *P. australis* extract was used as the extracting master batch, with a mass concentration of 100 g·L^−1^. For different parts of the aqueous extracts of *P. australis*, the sample was weighed accurately at 10 g, put into a conical flask, 100 ml of distilled water was added, sealed with a sealing strip, and soaked for 48 h. Then the conical flask was put into a shaker at the speed of 150 r/min and shaken intermittently during soaking. After the soaking, two layers of gauze were used to filter the large residues. The gauze was wrought as much as possible to collect a large amount of solution. For a second filtration, the solution from the previous step was loaded into a centrifuge tube and placed in a centrifuge at 2,500 r/min for 15 min, thus, the supernatant obtained was the extracts at a concentration of 100 g·L^−1^. All solutions were stored at 4°C.

### Germination experimental design

Germination tests were carried out in growth chambers with 16 h light and 8 h dark, at 25°C during the light period (Sylvania cool white fluorescent lamps, 200 μmol m^−2^ s^−1^, 400–700 nm) and 15°C during the dark period, with a relative air humidity of 75%. Seeds were germinated in a 9 cm diameter petri dish containing two layers of filter paper with 12 ml of test solution. Each petri dish was sealed with parafilm to prevent evaporation. Fifty selected *S. salsa* seeds of full and uniform size were placed evenly on the filter paper after the filter paper had been completely soaked with test solution or water as control, and four replicates of petri dishes were set up for each treatment. Seeds were considered germinated when the radicle protruded 1 mm from seed coat. Germination was recorded daily for 10 days.

To examine the interactive effects of different concentrations of salt stress, different concentrations of *P. australis* extract, and extracts of different organs of *P. australis* were combined with four salt concentrations (0, 100, 300, and 500 mM L^−1^ NaCl solution). Two concentrations of *P. australis* extract (50 and 6.25 g·L^−1^), and four parts of the *P. australis* community with belowground biomass (rhizome and root), aboveground biomass (shoot and leaf), litter (collected from the ground), and rhizosphere soil were used as different treatments.

After the germination period, the germination was evaluated, seedlings were collected and five seedlings were randomly selected from each replicate and radicle length was measured using a ruler. The seedlings were then wrapped and placed in an oven, dried at 60°C to constant weight and the dry weight was recorded.

Germination percentage (%) was calculated as the number of seeds that germinated in each solution per total number of seeds tested in in each solution, multiplied with 100. Germination rate was calculated as follows:


∑G/t×100%

where G is the daily germination rate and *t* is the total germination time (Khan and Ungar, 1984). The allelopathy was calculated by reference to the response index (*RI*; Williamson and Richardson, 1988)


$$RI=\left(T-T0\right)/T0$$

where T is the treatment value of the test item and T0 is the control value. If *RI > 0*, the effect is facilitative; if *RI < 0*, there is an inhibition effect, and the absolute value of *RI* represents the intensity of the effect.

### Data analysis

Statistical analysis of data was conducted by SPSS (version 25.0, SPSS Inc., Chicago, Illinois, United States). One-way ANOVA were used to confirm the effects of different extracts of *P. australis* organs on seed germination of *S. salsa* under the same salt concentration and the effects of different salt concentrations on seed germination of *S. salsa* under same organs. Duncan comparison tests were used to compare the differences between different treatments at the 0.05 level. Three-way ANOVA was used to analyze the effect among salt concentration, extract concentration, and extract organ, and the interaction effects between different treatments on seed germination and seedling growth. All acquired data were represented by an average of four replicates and standard deviation (SD).

## Results

### Germination performance

**Figure 3:**
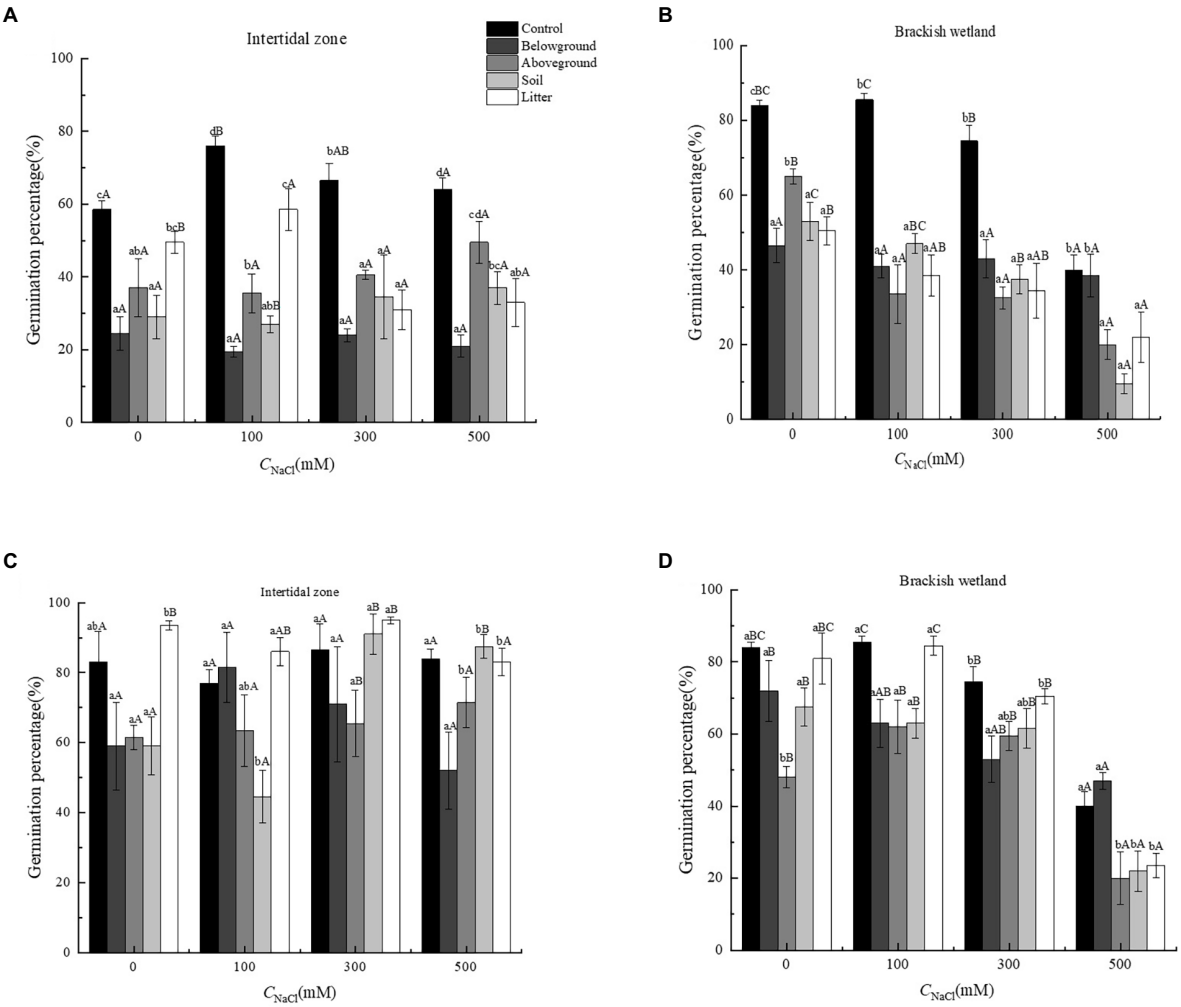
Effects of water extracts of *Phragmites australis* on the germination percentage of *Suaeda salsa* in different salt concentrations. **(A)** Germination percentage of *S. salsa* in intertidal zone under 50 g·L^−1^ *P. australis* extracts. **(B)** Germination percentage of *S. salsa* in brackish wetland under 50 g·L^−1^ *P. australis* extracts. **(C)** Germination percentage of *S. salsa* in intertidal zone under 6.25 g·L^−1^ *P. australis* extracts. **(D)** Germination percentage of *S. salsa* in brackish wetland under 6.25 g·L^−1^ *P. australis* extracts. Different lowercase letter means significant differences between different extracts of organs under the same salt concentration (*p* < 0.05); different capital letter means significant differences between different salt concentrations in the same extracts of organ (*p* < 0.05).

**Figure 4:**
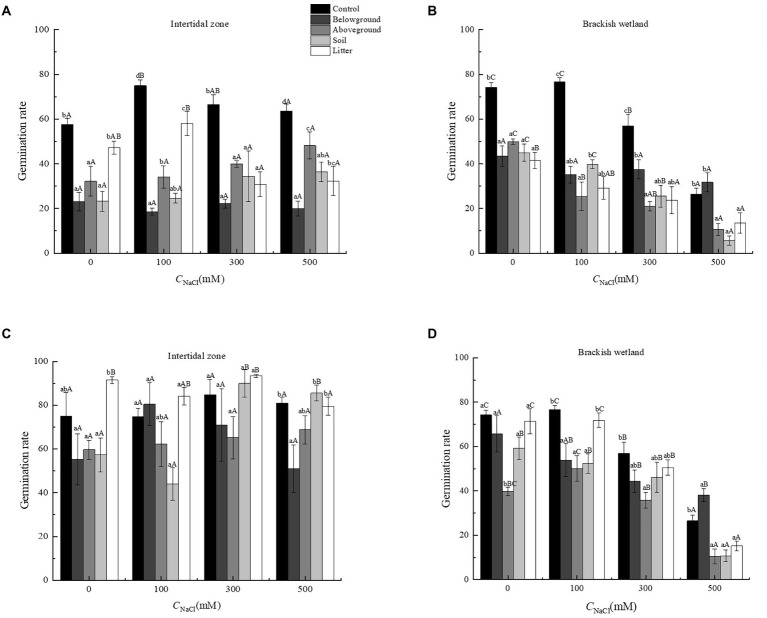
Effects of water extracts of *Phragmites australis* on the germination rate of *Suaeda salsa* in different salt concentrations. **(A)** Germination rate of *S. salsa* in intertidal zone under 50 g·L^−1^ *P. australis* extracts. **(B)** Germination rate of *S. salsa* in brackish wetland under 50 g·L^−1^ *P. australis* extracts. **(C)** Germination rate of *S. salsa* in intertidal zone under 6.25 g·L^−1^ *P. australis* extracts. **(D)** Germination rate of *S. salsa* in brackish wetland under 6.25 g·L^−1^ *P. australis* extracts. Different lowercase letter means significant differences between different extracts of organs under the same salt concentration (*p* < 0.05); different capital letter means significant differences between different salt concentrations in the same extracts of organ (*p* < 0.05).

The extracts of different organs of *P. australis* had significant negative effects on the germination percentage and germination rate of *S. salsa* from two different habitats (*p* < 0.05). At 6.25 g·L^−1^ extract concentration, only extracts of aboveground organs had a significant negative effect on the germination performance of *S. salsa*, relative to the control (Figures 3, 4). At 50 g·L^−1^ extract concentration, the extract of four different parts (belowground, aboveground, litter, and soil) significantly decreased the *S. salsa* seed germination percentage and germination rate of both intertidal zone and brackish wetland (*RI* < 0, Figures 3, 4; Tables 1, 2). Compared with the control treatment, the germination percentage and germination rate of *S. salsa* from the intertidal zone was lower in the different extract treatments (*p* < 0.05). Salt stress significantly affected the germination percentage and germination rate of *S. salsa* from a brackish wetland, but no significant effect was observed on seeds from the intertidal zone (Figures 3, 4). Three-way ANOVA showed that interaction of extract concentration and salt concentration had no significant effect on germination percentage or germination rate of *S. salsa* from the intertidal zone, but it did have a significant effect on *S. salsa* from a brackish wetland. The interaction of extract organ and salt concentration had a significant effect on germination of *S. salsa* from both habitats (Table 3).

**Table 1:** Response index (mean ± SE) of *S. salsa* in the intertidal zone under extract from different organs of *P. australis* and different salt concentrations.

Extract concentration		6.25 g·L^−1^				50 g·L^−1^			
Items	NaCl (mM)	Response index							
		Belowground	Aboveground	Soil	Litter	Belowground	Aboveground	Soil	Litter
Germination percentage	0	−0.23 ± 0.22	−0.24 ± 0.05	−0.23 ± 0.20	0.17 ± 0.13	−0.59 ± 0.07	−0.37 ± 0.14	−0.51 ± 0.09	−0.15 ± 0.04
	100	0.08 ± 0.17	–0.18 ± 0.12	–0.41 ± 0.13	0.13 ± 0.11	−0.74 ± 0.02	−0.53 ± 0.08	−0.64 ± 0.04	−0.23 ± 0.07
	300	–0.21 ± 0.16	–0.21 ± 0.1	0.09 ± 0.15	0.13 ± 0.11	−0.63 ± 0.04–	−0.38 ± 0.05	−0.49 ± 0.15	−0.53 ± 0.08
	500	–0.37 ± 0.14	–0.15 ± 0.08	0.04 ± 0.04	–0.01 ± 0.06	0.68 ± 0.04	–0.23 ± 0.06	–0.41 ± 0.09	–0.48 ± 0.10
Germination rate	0	−0.16 ± 0.25	−0.17 ± 0.09	−0.15 ± 0.24	0.30 ± 0.19	−0.60 ± 0.07	−0.44 ± 0.12	−0.61 ± 0.06	−0.18 ± 0.05
	100	0.10 ± 0.17	–0.16 ± 0.13	–0.40 ± 0.13	0.14 ± 0.12	–0.75 ± 0.03	–0.54 ± 0.08	–0.67 ± 0.03	–0.23 ± 0.07
	300	–0.20 ± 0.16	–0.20 ± 0.17	0.10 ± 0.16	0.13 ± 0.11	–0.66 ± 0.04	–0.39 ± 0.05	–0.50 ± 0.15	–0.53 ± 0.08
	500	–0.36 ± 0.15	–0.15 ± 0.08	0.06 ± 0.04	–0.01 ± 0.06	–0.69 ± 0.04	–0.25 ± 0.06	–0.42 ± 0.08	–0.49 ± 0.10
Radicle length	0	0.01 ± 0.10	0.25 ± 0.08	0.28 ± 0.07	0.36 ± 0.06	−0.81 ± 0.02	−0.15 ± 0.07	−0.13 ± 0.07	0.06 ± 0.13
	100	–0.01 ± 0.03	0.14 ± 0.06	0.20 ± 0.06	0.22 ± 0.05	–0.81 ± 0.00	–0.28 ± 0.03	0.05 ± 0.07	0.16 ± 0.04
	300	–0.07 ± 0.09	0.04 ± 0.03	0.18 ± 0.06	0.33 ± 0.02	–0.74 ± 0.02	–0.22 ± 0.12	–0.06 ± 0.04	0.22 ± 0.11
	500	0.05 ± 0.05	–0.04 ± 0.03	0.11 ± 0.05	0.65 ± 0.11	–0.74 ± 0.01	–0.29 ± 0.13	0.14 ± 0.18	0.02 ± 0.12
Biomass	0	−0.30 ± 0.07	−0.14 ± 0.07	−0.22 ± 0.06	−0.04 ± 0.07	−0.48 ± 0.09	0.17 ± 0.14	−0.11 ± 0.18	0.09 ± 0.16
	100	–0.24 ± 0.03	–0.14 ± 0.06	–0.25 ± 0.07	–0.05 ± 0.05	–0.68 ± 0.06	–0.26 ± 0.05	–0.20 ± 0.05	0.07 ± 0.04
	300	–0.36 ± 0.04	–0.17 ± 0.03	–0.27 ± 0.04	–0.10 ± 0.07	–0.61 ± 0.06	–0.33 ± 0.08	0.05 ± 0.09	0.14 ± 0.07
	500	–0.39 ± 0.03	–0.08 ± 0.06	–0.19 ± 0.01	0.02 ± 0.08	–0.64 ± 0.05	–0.24 ± 0.07	–0.08 ± 0.13	–0.11 ± 0.10

**Table 2:** Response index (mean ± SE) of *S. salsa* in the brackish wetland under extract from different organs of *P. australis* and different salt concentrations.

Extract concentration		6.25 g·L^−1^				50 g·L^−1^			
Items	NaCl (mM)	Response Index							
		Belowground	Aboveground	Soil	Litter	Belowground	Aboveground	Soil	Litter
Germination percentage	0	−0.14 ± 0.11	−0.43 ± 0.04	−0.20 ± 0.06	−0.04 ± 0.08	−0.45 ± 0.06	−0.23 ± 0.03	−0.37 ± 0.07	−0.40 ± 0.05
	100	–0.27 ± 0.06	–0.28 ± 0.08	–0.26 ± 0.05	–0.01 ± 0.04	–0.52 ± 0.03	–0.61 ± 0.10	–0.45 ± 0.04	–0.55 ± 0.06
	300	–0.28 ± 0.11	–0.20 ± 0.06	–0.17 ± 0.06	–0.04 ± 0.08	–0.42 ± 0.05	–0.55 ± 0.07	–0.50 ± 0.04	–0.54 ± 0.09
	500	0.19 ± 0.05	–0.53 ± 0.13	–0.47 ± 0.08	–0.41 ± 0.07	–0.13 ± 0.06	–0.48 ± 0.13	–0.53 ± 0.19	–0.48 ± 0.10
Germination rate	0	−0.11 ± 0.13	−0.46 ± 0.03	−0.20 ± 0.08	−0.04 ± 0.06	−0.41 ± 0.07	−0.33 ± 0.02	−0.39 ± 0.06	−0.44 ± 0.05
	100	–0.30 ± 0.08	–0.35 ± 0.07	–0.32 ± 0.05	–0.06 ± 0.06	–0.54 ± 0.05	–0.67 ± 0.09	–0.48 ± 0.04	–0.62 ± 0.06
	300	–0.20 ± 0.12	–0.35 ± 0.09	–0.18 ± 0.10	–0.08 ± 0.14	–0.33 ± 0.07	–0.61 ± 0.08	–0.56 ± 0.06	–0.57 ± 0.11
	500	0.44 ± 0.03	–0.63 ± 0.09	–0.61 ± 0.06	–0.42 ± 0.09	0.22 ± 0.18	–0.58 ± 0.13	–0.77 ± 0.09	–0.51 ± 0.11
Radicle length	0	−0.61 ± 0.00	−0.39 ± 0.05	−0.14 ± 0.08	−0.06 ± 0.04	−0.73 ± 0.02	−0.36 ± 0.01	−0.15 ± 0.05	0.13 ± 0.07
	100	–0.60 ± 0.04	–0.24 ± 0.07	–0.09 ± 0.05	0.00 ± 0.03	–0.76 ± 0.02	–0.04 ± 0.05	0.00 ± 0.06	0.03 ± 0.06
	300	–0.57 ± 0.03	0.04 ± 0.08	0.23 ± 0.15	0.17 ± 0.07	–0.71 ± 0.03	0.17 ± 0.12	0.17 ± 0.12	0.40 ± 0.15
	500	–0.50 ± 0.03	–0.20 ± 0.07	–0.14 ± 0.05	0.45 ± 0.05	–0.54 ± 0.05	0.51 ± 0.07	0.10 ± 0.12	0.54 ± 0.04
Biomass	0	−0.02 ± 0.12	−0.28 ± 0.07	−0.03 ± 0.18	0.33 ± 0.18	−0.47 ± 0.11	−0.36 ± 0.03	−0.01 ± 0.11	0.42 ± 0.19
	100	–0.41 ± 0.07	–0.33 ± 0.02	–0.19 ± 0.09	0.05 ± 0.22	–0.71 ± 0.03	–0.37 ± 0.06	–0.17 ± 0.08	–0.19 ± 0.13
	300	–0.38 ± 0.10	–0.24 ± 0.09	0.31 ± 0.29	0.19 ± 0.16	–0.50 ± 0.09	–0.12 ± 0.09	0.28 ± 0.15	0.04 ± 0.13
	500	–0.25 ± 0.15	–0.19 ± 0.15	0.12 ± 0.12	0.09 ± 0.12	–0.41 ± 0.15	–0.07 ± 0.14	–0.22 ± 0.20	–0.13 ± 0.12

**Table 3:** Three-way ANOVA of the response of different stress factors to the seed germination and growth of *S. salsa* in the intertidal zone.

Index	Germination percentage		Germination rate		Radicle length		Biomass	
	*F*	*P*	*F*	*P*	*F*	*P*	*F*	*P*
Intertidal zone								
Extract concentration (EC)	259.917	**0.000**	255.957	**0.000**	263.230	**0.000**	92.962	**0.000**
Salt concentration (SC)	1.058	0.370	2.254	**0.000**	14.915	**0.000**	44.580	**0.000**
Organ	27.113	**0.000**	26.793	**0.000**	13.267	**0.000**	95.911	**0.000**
EC × SC	2.350	0.076	2.326	0.078	13.537	**0.000**	2.217	0.090
EC × Organ	7.443	**0.000**	8.591	**0.000**	19.518	**0.000**	13.535	**0.000**
SC × Organ	3.146	**0.001**	3.380	**0.000**	31.071	**0.005**	2.866	**0.002**
EC × SC × Organ	1.857	**0.047**	1.700	0.075	35.627	**0.000**	2.869	**0.002**
Brackish wetland								
Extract concentration (EC)	87.826	**0.000**	77.705	**0.000**	12.118	**0.001**	8.597	**0.004**
Salt concentration (SC)	116.529	**0.000**	153.108	**0.000**	44.450	**0.000**	3.883	**0.000**
Organ	43.226	**0.004**	50.865	**0.000**	279.291	**0.000**	32.768	**0.000**
EC × SC	7.264	**0.000**	7.041	**0.006**	2.880	**0.039**	0.575	0.633
EC × Organ	9.457	**0.000**	9.585	**0.000**	12.656	**0.000**	2.951	**0.023**
SC × Organ	3.735	**0.000**	4.642	**0.003**	10.452	**0.000**	2.880	**0.002**
EC × SC × Organ	3.300	**0.000**	2.690	**0.003**	2.289	**0.012**	0.921	0.529

Significant effects (*p* < 0.05) are in bold.

### Radicle length

The extracts of different organs of *P. australis* had negative effects on the radicle length of *S. salsa* seedlings from the two habitats (Table 4). 6.25 g·L^−1^ belowground extract significantly decreased the radicle length of *S. salsa* seedlings from the brackish wetland (*RI* < 0, Tables 2, 4), but did not significantly affect the seedlings from the intertidal zone (Table 4). At 50 g·L^−1^ extract concentration, belowground extract significantly decreased the radicle length of *S. salsa* seedlings from both habitats (*p* < 0.05, *RI* < 0, Tables 1, 2, 4). However, litter extract significantly increased the radicle length of the seedlings at 100 and 300 mM NaCl concentration (intertidal zone; *RI* > 0, Tables 1, 4), and at 0, 300, and 500 mM NaCl concentration (brackish wetland; *RI* > 0, Tables 2, 4). The response index of *S. salsa* from the intertidal zone showed that 6.25 g·L^−1^ extract concentration of different organs promoted or slightly inhibited the radicle length (Table 1), but 50 g·L^−1^ extract concentration of belowground and aboveground part strongly inhibited the radicle length (*RI* < 0, Table 1). While the response index of *S. salsa* from the brackish wetland showed that belowground extracts of both 6.25 and 50 g·L^−1^ inhibited the radicle length (*RI* < 0, Table 2). With increasing salt concentration, the radicle length decreased significantly (Table 4, *p* < 0.05). Three-way ANOVA showed that there was a significant interaction of extract concentration, salt concentration, and extract organ on the radicle length of *S. salsa* seedlings of the two habitats (Table 3).

**Table 4:** Effects of water extracts of *P. australis* on the radicle length of *S. salsa* in different salt concentrations (mean ± SE).

		Intertidal zone		Brackish wetland	
NaCl (mM)	Organ	Radicle length (cm)			
		50 g·L^-1^	6.25 g·L^-1^	50 g·L^-1^	6.25 g·L^-1^
0	Control	5.99 ± 0.24cA	5.34 ± 0.18aA	4.67 ± 0.13dA	4.67 ± 0.13dA
	Belowground	1.11 ± 0.03aA	5.28 ± 0.17aA	1.25 ± 0.05aAB	1.80 ± 0.06aAB
	Aboveground	5.05 ± 0.22bA	6.58 ± 0.13bA	2.98 ± 0.14bA	2.83 ± 0.12bB
	Soil	5.23 ± 0.32bA	6.78 ± 0.13bcA	3.94 ± 0.15cAB	4.02 ± 0.15cB
	Litter	6.21 ± 0.21cA	7.19 ± 0.12cA	5.29 ± 0.19eA	4.39 ± 0.16cdA
100	Control	5.87 ± 0.13cA	5.26 ± 0.11aA	4.88 ± 0.12bA	4.88 ± 0.12dA
	Belowground	1.10 ± 0.02aA	5.22 ± 0.10aA	1.16 ± 0.07aAB	1.95 ± 0.09aA
	Aboveground	4.21 ± 0.22bB	6.00 ± 0.13bB	4.66 ± 0.20bB	3.69 ± 0.17bC
	Soil	6.16 ± 0.31cB	6.28 ± 0.12bcB	4.87 ± 0.13bC	4.45 ± 0.17cC
	Litter	6.81 ± 0.22dA	6.42 ± 0.12cB	5.04 ± 0.20bA	4.88 ± 0.13 dB
300	Control	4.64 ± 0.09cB	5.11 ± 0.08abA	3.84 ± 0.16bB	3.84 ± 0.16bB
	Belowground	1.22 ± 0.05aB	4.74 ± 0.17aB	1.09 ± 0.07aA	1.62 ± 0.07aBC
	Aboveground	3.64 ± 0.29bB	5.35 ± 0.12bC	4.39 ± 0.12cB	3.92 ± 0.13bC
	Soil	4.38 ± 0.30cA	5.98 ± 0.12cA	4.43 ± 0.19cBC	4.62 ± 0.17cC
	Litter	5.68 ± 0.27 dB	6.83 ± 0.15 dB	5.27 ± 0.19dA	4.42 ± 0.17cA
500	Control	4.19 ± 0.18cB	4.10 ± 0.08aB	2.93 ± 0.10bC	2.93 ± 0.10cC
	Belowground	1.07 ± 0.03aA	4.28 ± 0.11abC	1.35 ± 0.03aB	1.45 ± 0.06aC
	Aboveground	2.87 ± 0.20bC	3.95 ± 0.10aD	4.41 ± 0.30cB	2.35 ± 0.16bA
	Soil	4.61 ± 0.30cA	4.56 ± 0.10bC	3.15 ± 0.53cA	2.50 ± 0.12bA
	Litter	4.14 ± 0.20cC	6.72 ± 0.19cA	4.50 ± 0.17cB	4.25 ± 0.13cA

Different lowercase letter means significant differences between different extracts of organs under the same salt concentration (*p* < 0.05); different capital letter means significant differences between different salt concentrations in the same extracts of organ (*p* < 0.05).

### Seedling biomass

The extracts from different parts of *P. australis* had different effects on the seedling biomass of *S. salsa* from the two habitats (Table 5). Among them, belowground extract significantly decreased the seedling biomass of *S. salsa* from the two habitats (*p* < 0.05). With increasing salt concentration, the seedling biomass from the intertidal zone increased significantly within the extracts of different parts (*p* < 0.05), except at 50 g·L^−1^ belowground and aboveground extracts (Table 5). The seedling biomass of *S. salsa* from a brackish wetland significantly increased at 50 g·L^−1^ soil extract and 300 mM NaCl (*RI* > 0, Table 2). However, at 50 g·L^−1^ aboveground extracts, all three NaCl concentration (100, 300, and 500 mM) significantly decreased the seedling biomass (*p* < 0.05*, RI* < 0, Tables 2, 5). The salt concentration of 300 mM NaCl with soil extract at 6.25 g·L^−1^ significantly increased the seedling biomass when compared with 0 salt treatment (*p* < 0.05).

**Table 5:** Effects of water extracts of *P. australis* on the biomass of *S. salsa* in different salt concentrations (mean ± SE).

		Intertidal zone		Brackish wetland	
NaCl (mM)	Organ	Biomass(mg/plant)			
		50 g·L^-1^	6.25 g·L^-1^	50 g·L^-1^	6.25 g·L^-1^
0	Control	1.38 ± 0.11bcA	1.79 ± 0.12dA	0.68 ± 0.07bA	0.68 ± 0.07abA
	Belowground	0.69 ± 0.09aA	1.25 ± 0.08aA	0.34 ± 0.06aA	0.67 ± 0.11abA
	Aboveground	1.58 ± 0.09cA	1.52 ± 0.05bcA	0.43 ± 0.02acA	0.48 ± 0.04aA
	Soil	1.18 ± 0.16bA	1.38 ± 0.03abA	0.65 ± 0.04bA	0.64 ± 0.09abA
	Litter	1.45 ± 0.08bcA	1.70 ± 0.08cdA	0.92 ± 0.06cA	0.89 ± 0.06bA
100	Control	1.94 ± 0.04cB	2.03 ± 0.07cA	0.97 ± 0.05cB	0.97 ± 0.05bB
	Belowground	0.62 ± 0.13aA	1.53 ± 0.06bA	0.28 ± 0.03aA	0.57 ± 0.07aA
	Aboveground	1.43 ± 0.08bA	1.74 ± 0.10abA	0.61 ± 0.05bB	0.65 ± 0.03aA
	Soil	1.55 ± 0.09bAB	1.50 ± 0.09aA	0.81 ± 0.12bcAB	0.78 ± 0.08abAB
	Litter	2.08 ± 0.06cB	1.94 ± 0.11bcAB	0.77 ± 0.09bcA	0.99 ± 0.18bA
300	Control	1.93 ± 0.11cB	2.44 ± 0.09 dB	0.79 ± 0.07bcAB	0.79 ± 0.07bcAB
	Belowground	0.73 ± 0.09aA	1.56 ± 0.11aB	0.38 ± 0.05aA	0.49 ± 0.11aA
	Aboveground	1.29 ± 0.15bA	2.04 ± 0.07bcB	0.68 ± 0.05bB	0.58 ± 0.02abA
	Soil	2.00 ± 0.12cB	1.78 ± 0.12abB	0.98 ± 0.10cB	0.98 ± 0.14cB
	Litter	2.18 ± 0.10cB	2.20 ± 0.10cdBC	0.81 ± 0.09bcA	0.91 ± 0.06cA
500	Control	2.17 ± 0.14bB	2.47 ± 0.10cB	0.85 ± 0.08bAB	0.85 ± 0.08abAB
	Belowground	0.78 ± 0.11aA	1.50 ± 0.03aB	0.47 ± 0.09aA	0.62 ± 0.11aA
	Aboveground	1.66 ± 0.22bA	2.26 ± 0.12abB	0.77 ± 0.08bB	0.67 ± 0.11abA
	Soil	1.96 ± 0.18bB	2.00 ± 0.07bB	0.64 ± 0.12abA	0.93 ± 0.08bAB
	Litter	1.88 ± 0.15bB	2.51 ± 0.17cC	0.72 ± 0.05abA	0.90 ± 0.04abA

Different lowercase letter means significant differences between different extracts of organs under the same salt concentration (*p* < 0.05); different capital letter means significant differences between different salt concentrations in the same extracts of organ (*p* < 0.05).

The response index of *S. salsa* from the intertidal zone showed that 6.25 g·L^−1^ extract concentration of different organs inhibited the seedling biomass (*RI* < 0, Table 1), expect the litter extract at the 500 mM NaCl treatment (*RI* > 0, Table 1). However, at 50 g·L^−1^ extract concentration, only the belowground extract inhibited the seedling biomass (*RI* < 0, Table 1). The effects of extract of other organs on seedling biomass were different under different salt concentrations (Table 5). For the brackish wetland, both 6.25 and 50 g·L^−1^ extract concentrations of belowground and aboveground parts inhibited the seedling biomass (*RI* < 0, Table 2), but 6.25 g·L^−1^ litter extracts in all salt concentrations promoted the seedling biomass (*RI* > 0, Table 2). Similarly, Three-way ANOVA showed that there was no interaction of extract concentration and salt concentration on seedling biomass of *S. salsa* from the two different habitats. However, there was a significant interaction of extract organ and salt concentration on seedling biomass, and the inhibition was highest for the belowground extract (Table 3).

## Discussion

This study demonstrated that aqueous extracts from aboveground and belowground biomass, soil, and litter of *P. australis* exhibited allelopathic activity on *S. salsa* seed germination and seedling growth, while the strength of the response depended on the habitat of the plants. Especially in halophytes, different habitat conditions can cause seed dimorphism and polymorphism (Khan et al., 2001; Zhang et al., 2021). In the present study, the seed weight of the intertidal zone was greater than that of the brackish wetland. In certain plant species, seed size can positively affect germination and seedling vigor under stressful conditions, since they have higher amounts of starch and other energy reserves needed to counteract the stress (Mut and Akay, 2010; Steiner et al., 2019). In particular, salt stress decreased the germination percentage and germination rate of *S. salsa* of the brackish wetland, but had no significant effect on the seed germination from the intertidal zone. Adaptation to a saline habitat that is the intertidal zone is a likely explanation for our observation (Song et al., 2008). Locally adapted seeds are prone to withstand the harsh conditions of their provenance and preferably used for restoration purposes (Hufford and Mazer, 2003; Broadhurst et al., 2008). Hence, the intertidal ecotype responded to a lower degree to salt stress than did the brackish ecotype.

Previous studies on *S. salsa* from brackish wetlands and other halophytes showed that plant height decreased with the increasing salt stress, but biomass production was stimulated at low salinities and only decreased with more intense salt stress (Guan et al., 2013; Parida and Jha, 2014; Parida et al., 2016). In this study, similar trends on radicle length and seedling biomass were observed for the seedlings from both the brackish wetland and the intertidal zone. Generally, when plants encounter salt stress, some growth indicators will decrease (Saddiq et al., 2021), but halophytes, such as *S. salsa*, will invest in longer, thinner roots to gain better access to inorganic nutrients (Wang et al., 2021). In agreement with this, the intertidal ecotype had generally longer roots, and reduced its radicle length to a lesser degree than the brackish ecotype with increasing salinity.

The addition of *P. australis* extract, especially from belowground parts, had an overall stronger inhibitory effect on the seed germination and seedling growth at the higher concentration of extract solution (50 g·L^−1^), compared to the low concentration (6.25 g·L^−1^), as could be expected (Tefera, 2002; Bogatek et al., 2006). One aspect of the high inhibition effect of belowground extract could be due to a higher concentration of phenolic acid compounds in the roots of *P. australis*, which suppress the activity of α-amylase that plays an important role for successful seed germination (Uddin et al., 2017; Liu et al., 2018). More importantly, the germination rate, percentage, and radicle length of brackish ecotypes were significantly affected by an interaction of *P. australis* extracts and salinity. The intertidal zone ecotypes also had an interaction, although only with respect to radicle length. For both ecotypes, the negative response to increasing salinity was stronger when exposed to the high extract concentration, while the control with zero extract stayed similar. Hence, allelopathy can potentially worsen abiotic stresses, although it has been shown that high soil salinity can also lower allelopathic substances due to a reduction of secondary metabolite production in the source plant (Kotzamani et al., 2021). Weeds and invasive plants have been shown to be quite salt tolerant when compared to crops (Cirillo et al., 2018). *Phragmites australis* often behaves like a weed, with its high intraspecific genetic variation, remarkable phenotypic plasticity, ecological niche breadth, and high productivity (Eller et al., 2017). A salt-tolerant weed like *P. australis* can be expected to have augmented inhibitory effects by enhanced phytotoxicity of its allelochemicals under high salt concentrations (Khalid et al., 2002; El-Darier and Youssef, 2017; Cirillo et al., 2018), as we observed here. In salt-tolerant plants, enzymatic activity associated with reactive oxygen species (ROS) scavenging is high, as a means of stress defense (Munns and Tester, 2008). Although allelochemicals can suppress specific scavenging enzymes, the antioxidant defense system of salt-tolerant plants may be constituted to counteract the harmful effects of ROS generated during seed germination and initial growth in the presence of toxic allelochemicals (Coelho et al., 2017).

Few studies have compared the effects of allelopathic extracts from different parts of the plant community. Among all the extracts, the belowground and aboveground parts showed the overall strongest inhibition, which were well aligned with previous studies of phytotoxic evaluation of *P. australis* (Uddin et al., 2012). *Phragmites australis* is a persistent grass with a long rootstock (rhizome) and high growth rates. The rhizomes and roots in soil keep their viability for many years and the robust rhizome is of great importance in the life of this plant (Uddin and Robinson, 2017). In this study, the strong allelopathic effect of belowground extracts could be another important reason for its high competitiveness to other, coexisting species. *Phragmites australis* root secretions contain gallic acid, which induces the production of reactive oxygen species and disrupts plant root growth by causing peroxidation of membrane lipids and membrane damage, thereby impeding neighboring plant growth and development (Rudrappa et al., 2007).

Interestingly, not all the extracts of *P. australis* showed allelopathic effects on the radicle length and seedling biomass. The low concentration of litter extract significantly promoted seedling radicle length of the intertidal zone ecotype, especially in higher salt concentrations, suggesting that nutrient elements in the extract could provide some nutrients for the seedling growth.

## Conclusion

We found that the extracts of different organs of *P. australis*, with the strongest inhibition caused by belowground and aboveground extracts, had an allelopathic effect on the germination and growth of *S. salsa* from two habitats. With increasing salt stress, the germination performance of the seeds from the intertidal zone showed high salt tolerance, but seeds from the brackish wetland showed salt stress, which was enhanced by the allelochemical extracts. Our study suggests that allelopathy is one of the important reasons of high competitiveness and invasive capacity of *P. australis* in its habitats. As the allelopathic potential was mainly exerted at higher extract concentrations, we suggest that allelopathic chemicals should be analyzed in natural soils, where *P. australis* and *S. salsa* co-occur. Moreover, it is possible that extracts from reed ecotypes differ, in addition to our observation of different stress tolerance of *S. salsa* ecotypes. Cross-testing of *P. australis* extracts from different provenances with *S. salsa* ecotypes could provide insights for this assumption. *Phragmites australis* is commonly used for restoration in Chinese wetlands. Understanding its allelochemical effects is crucial to avoid undesired inhibition of coexisting flora. This study represents a valuable step forward in our understanding of allelopathic effects from different parts of the *P. australis* community.

## Data availability statement

The original contributions presented in the study are included in the article/supplementary material, further inquiries can be directed to the corresponding authors.

## Author contributions

BG and JZ designed the study. JG and MG conducted the control experiment. JG and BG carried out the data analysis and wrote the manuscript. FE, JY, and XW revised the manuscript. BG, XW, and JY coordinated the project. All authors contributed to the article and approved the submitted version.

## Funding

This work was financially supported by the National Natural Science Foundation of China (41871091, 42171111, and U1806218).

## Conflict of interest

The authors declare that the research was conducted in the absence of any commercial or financial relationships that could be construed as a potential conflict of interest.

## Publisher’s note

All claims expressed in this article are solely those of the authors and do not necessarily represent those of their affiliated organizations, or those of the publisher, the editors and the reviewers. Any product that may be evaluated in this article, or claim that may be made by its manufacturer, is not guaranteed or endorsed by the publisher.
